# Predictors of coronary artery injury after orbital atherectomy as assessed by optical coherence tomography

**DOI:** 10.1007/s10554-023-02837-7

**Published:** 2023-04-07

**Authors:** Tetsumin Lee, Takashi Ashikaga, Toshihiro Nozato, Masakazu Kaneko, Ryoichi Miyazaki, Shinichiro Okata, Masashi Nagase, Tomoki Horie, Mao Terui, Tetsuya Kishigami, Yasutoshi Nagata, Toru Misawa, Yuta Taomoto, Daigo Kachi, Michihito Naito, Taishi Yonetsu, Tetsuo Sasano

**Affiliations:** 1 Department of Cardiology, Japanese Red Cross Musashino Hospital, 1-26-1 Kyonancho, Musashinoshi, Tokyo, Tokyo Japan; 2 Department of Cardiovascular Medicine, Tokyo Medical and Dental University, Tokyo, Japan

**Keywords:** Calcified stenosis, Optical coherence tomography, Coronary artery disease, Orbital atherectomy

## Abstract

**Purpose:** The association between the extent of the wire and device bias as assessed by optical coherence tomography (OCT) in the healthy portion of the vessel and the risk of coronary artery injury after orbital atherectomy (OA) has not been fully elucidated. Thus, purpose of this study is to investigate the association between pre-OA OCT findings and post-OA coronary artery injury by OCT. **Methods:** We enrolled 148 de novo lesions having calcified lesion required OA (max Ca angle > 90°) in 135 patients who underwent both pre- and post-OA OCT. In pre-OA OCT, OCT catheter contact angle and the presence or absences of guide-wire (GW) contact with the normal vessel intima were assessed. Also, in post-OA OCT, we assessed there was post-OA coronary artery injury (OA injury), defined as disappearance of both of intima and medial wall of normal vessel, or not. **Results:** OA injury was found in 19 lesions (13%). Pre-PCI OCT catheter contact angle with the normal coronary artery was significantly larger (median 137°; inter quartile range [IQR] 113–169 vs. median 0°; IQR 0–0, P < 0.001) and more GW contact with the normal vessel was found (63% vs. 8%, P < 0.001). Pre-PCI OCT catheter contact angle > 92° and GW contact with the normal vessel intima were associated with post-OA vascular injury (Both: 92% (11/12), Either: 32% (8/25), Neither: 0% (0/111), P < 0.001). **Conclusion:** Pre-PCI OCT findings, such as catheter contact angle > 92° and guide-wire contact to the normal coronary artery, were associated with post-OA coronary artery injury.

## Introduction

Severely calcified coronary lesions are associated with poor outcomes and present a major challenge in patients undergoing percutaneous coronary intervention (PCI) due to high risk of stent thrombosis, target lesion failure, difficulty in stent delivery, and stent underexpansion [1]. In order to avoid the aforementioned complications, lesion preparation prior to PCI has become increasingly important for calcified coronary lesion. Orbital atherectomy (OA) (Cardiovascular Systems, Inc. St. Paul, MN, USA) is an atherectomy treatment option which utilizes centrifugal force, allowing for 360° contact of the vessel wall and an effective to treat the lesions with severally calcified plaque [2]. While the usefulness of OA for the treatment of calcified lesion, the past registry data reported that the prevalence of coronary perforation was reported as 0.7% at PCI with OA and the occurrence of coronary perforation results in poor outcomes despite of the rare complication events [3]. Although the mechanism of the coronary perforation after OA remains to be elucidated, excessive ablation by OA of the normal segment is reported to be a potential cause of coronary perforation and the risk of coronary perforation is considered to increase when the wire ant the OA device are in contact with the healthy portion of the vessel.

An optical coherence tomography (OCT) is a high-resolution imaging device, especially at lesions with calcified plaque. OCT enables us to assess quantitative analysis of calcium (Ca) plate, such as thickness, angle, and length, as well as the excessive ablation of the intima and media of the normal coronary artery segment [4, 5]. Wire and device bias as assessed by intravascular imaging was reported as one of the predictors of coronary artery injury after rotational atherectomy (RA) or OA in the past studies and case reports [6]. However, the association between the extent of wire and the device bias as assessed by OCT in the healthy portion of the vessel and the risk of coronary artery injury is still unclear. Therefore, the aim of the present study is to investigate the association pre-OA OCT findings and post-OA coronary artery injury as assessed by OCT.

## Methods

### Study population

This was a retrospective observational study at Japanese Red Cross Musashino Hospital (Tokyo, Japan). From April 2018 to June 2021, there were 159 calcified lesions (max Ca angle > 90° on OCT) in 145 patients who underwent OCT imaging during PCI with OA. We excluded 6 lesions without pre-OA OCT, 4 lesions without post-OA OCT, and 1 lesion with poor image quality. Finally, 148 de novo lesions with Ca angle ≥ 90° in 134 patients who underwent both pre- and post-OA OCT were enrolled in this study. Lesions with anticipated difficulty in advancing the OCT catheter, such as lesions with severe narrowing, tortuosity or severe calcification, were excluded and not imaged by the operators. This study was performed in line with the principles of the Declaration of Helsinki. Approval was granted by the Ethics Committee of Japanese Red Cross Musashino Hospital (Date: June 10, No 4026).

### Coronary angiographic analysis

Quantitative coronary angiography was performed using QCA-CMS (Medis medical imaging systems, Leiden, The Netherlands). Minimum lumen diameter, reference diameter, and lesion length were measured in diastolic frames from orthogonal projections. Angiographic calcification was classified as none or mild, moderate, or severe at the target lesion site [7]. Moderate calcification was defined as radio-opacities noted only during the cardiac cycle before contrast injection, whereas severe calcification was defined as radio-opacities seen without cardiac motion, usually affecting both sides of the arterial lumen. Maximum coronary artery angle was defined as the maximum angiographic angle using the view with maximum angle [4].

### OCT image acquisition and analysis

We used frequency-domain OCT (Dragonfly OPTIS or Opstar OCT imaging catheter, Abbott Vascular, Santa Clara, CA, USA) and all OCT images were analyzed using proprietary software using previously validated criteria for OCT plaque characterization [8, 9].

**Fig. 1:**
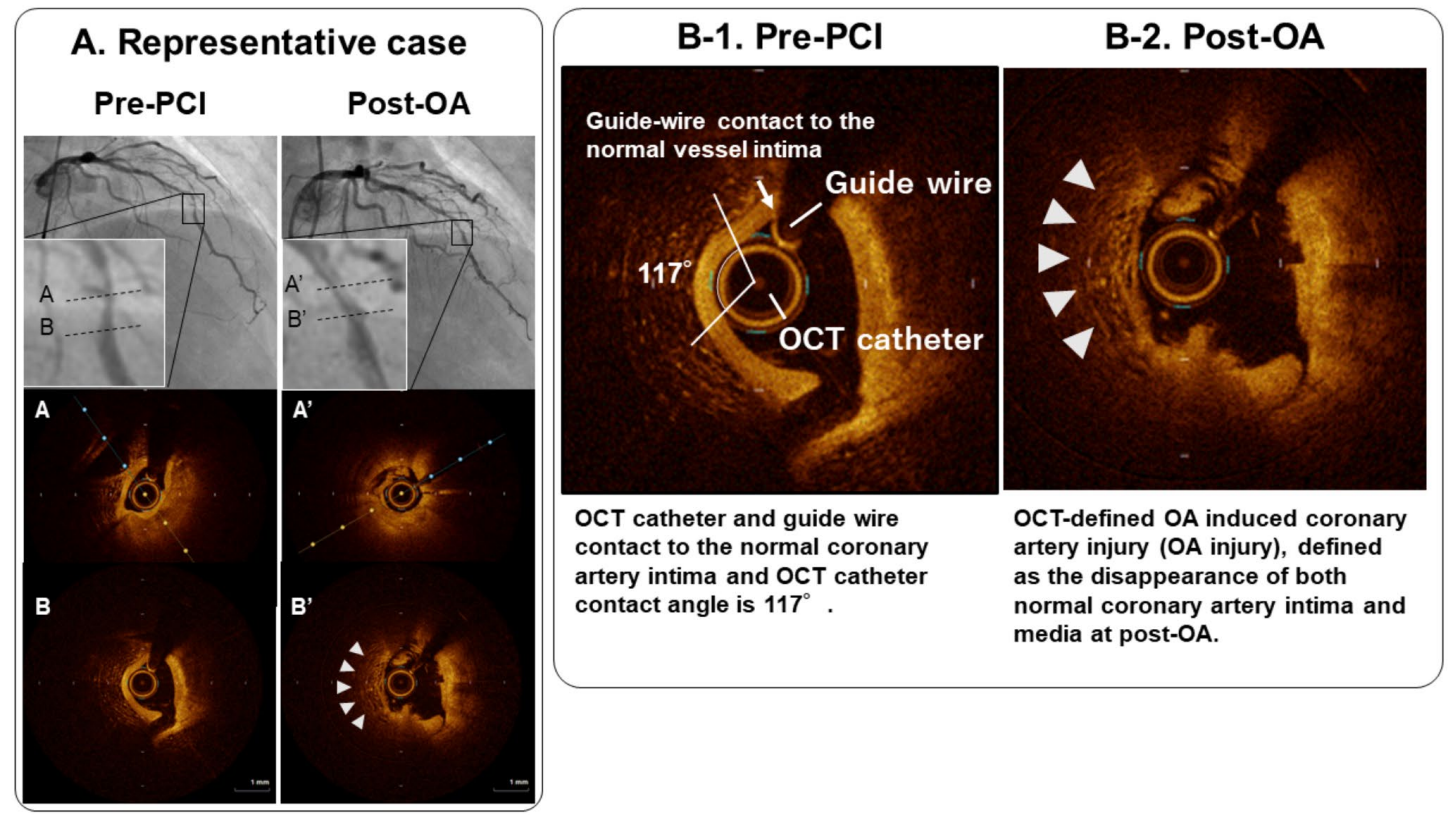
Representative Case of Orbital Atherectomy Injury and Magnified Image of Optical Coherence Tomography (A) In Pre-percutaneous coronary intervention (PCI) angiogram and optical coherence tomography (OCT), there are severely calcified plaque at mid left anterior descending (LAD) artery and post-orbital atherectomy (OA) OCT showed intima and media were disappeared at normal vessel segment (white arrow head). (B-1) Magnified OCT image at pre-PCI showed OCT catheter and guide wire contact to the normal coronary artery intima and OCT catheter contact angle is 117°. (B-2) Magnified image of post-OA OCT showed OCT-defined OA induced coronary artery injury (OA injury), defined as the disappearance of both normal coronary artery intima and media at post-OA (white arrow head)

We evaluated all cross-sectional OCT frames in which OA was performed at both pre- and post-OA. Calcium was defined as a signal-poor or heterogeneous region with a sharply delineated border. The maximum arc of target lesion calcium was measured in degrees with a protractor centered on the lumen. Maximum calcium thickness was also measured [10]. A calcified nodule was defined as an accumulation of small calcium deposits underlying calcified plate which includes either pathological eruptive calcified nodule or nodular calcification [4, 11]. At pre-OA OCT, to assess the device and wire bias, we checked whether OCT imaging catheter and guide-wire were in direct contact with the normal coronary artery wall [12]. In addition, for the quantitative analysis of device and wire bias, each measurement of OCT imaging was defined as follows; (1) OCT contact angle: the arc of contact between OCT imaging catheter and the intima of normal coronary artery wall; (2) OCT contact length: the longitudinal length of OCT imaging catheter to the normal coronary artery; (3) GW contact length: the longitudinal length of guide-wire (GW) to the normal coronary artery (Fig. 1). At post-OA OCT, we assessed the presence or absence of OA induced coronary artery injury (OA injury), defined as the disappearance of both normal vessel intima and media at post-OA due to OA debulking (Fig. 1).

### Statistical analysis

Data analysis was performed using SPSS 22.0 (IBM, Armonk, New York). Categorical data were expressed as frequencies and compared using the χ^2^ or Fisher exact test, as appropriate. The normality of the data was verified using the Kolmogorov-Smirnov test. Continuous variables were expressed as mean ± standard deviation for normally distributed variables and as median (first quartile, third quartile) for nonnormally distributed variables, and compared by use of the Student *t* test and Mann-Whitney *U* tests, respectively. Inter- and intra-observer variability were tested using intra-class correlation coefficient (ICC) for OCT contact angle, OCT contact length, and GW contact length. Inter-observer variability was assessed by two independent observers (S.O and D K) and intra-observer variability was assessed by reanalysis of a single observer 4 weeks later. The relationship between OA injury (dependent variable), clinical and angiographic characteristics, OCT findings, and other potential confounders was assessed using a univariable logistic regression analysis to determine whether the OCT findings remained associated with OA injury.

## Results

All patients underwent PCI procedure without severe complication required additional PCI treatment, including coronary perforation. There was a good concordance of inter- and intra-observer agreement for the measurement of OCT contact angle (ICC = 0.84, 0.93), OCT contact length (0.81, 0.90), and GW contact length (ICC = 0.81, 0.85), respectively.

### Clinical, angiographic, and PCI findings

Median patient age was 74 years (interquartile range [IQR]: 69–80), 17% were female, and 92% presented with stable angina (Table 1). We found OA injury in 19 lesions in 17 patients and there were no significant differences of patient characteristics between patients with or without OA injury. In angiographic and PCI findings, larger maximum coronary angle at lesion and less maximum OA speed of 120,000 were found in lesions with OA injury than in those without (Table 2), whereas there were no significant differences of other findings, including lesion location, pre-PCI QCA findings, classic crown OA device usage, and total OA ablation frequency and time. Although TIMI 0–2 coronary flow just after OA was found in 6 lesions (4%), slow or no-flow was not observed at final angiogram. In addition, there was no significant post-PCI angiographic dissection.

**Table 1:** Patient Characteristics

	Overall (n = 134)	Patients with OA injury (n = 17)	Patients without OA injury (n = 117)	*P*
Age, yrs	74.0 (69.0–80.0)	75.0 (69.7–78.3)	74.0 (68.8–81.0)	0.85
Male gender, % (n)	111 (82.8)	13 (76.5)	98 (83.8)	0.46
Stable angina, % (n)	123 (91.8)	15 (88.2)	108 (92.3)	0.63
Diabetes mellitus, % (n)	63 (47.0)	8 (47.1)	55 (47.0)	1.00
Hypertension, % (n)	112 (83.6)	14 (82.4)	98 (83.8)	0.88
Dyslipidemia, % (n)	94 (70.1)	9 (52.9)	85 (72.6)	0.15
Current smoker, % (n)	42 (31.3)	6 (35.3)	36 (30.8)	0.78
Previous myocardial infarction, % (n)	39 (29.1)	4 (23.5)	35 (29.9)	0.78
Previous PCI, % (n)	70 (52.2)	8 (47.1)	62 (53.0)	0.80
Previous CABG, % (n)	4 (3.0)	1 (5.9)	3 (2.6)	0.42
Renal insufficiency required Hemodialysis, % (n)	28 (20.9)	4 (23.5)	24 (20.5)	0.76

PCI indicates percutaneous coronary intervention; CABG, coronary artery bypass surgery

**Table 2:** Angiographic and Procedure Results

	Overall (n = 148)	Lesions with OA injury (n = 19)	Lesions without OA injury (n = 129)	*P*
Target vessel, % (n)				0.39
LAD	99 (66.9)	11 (57.9)	88 (68.2)	
LCX	12 (8.1)	3 (15.8)	9 (7.0)	
RCA	37 (25.0)	5 (26.3)	32 (24.8)	
Lesion location				0.20
Ostium	18 (12.2)	5 (26.3)	13 (10.1)	
Proximal	61 (41.2)	9 (47.4)	52 (40.3)	
Mid	63 (42.6)	5 (26.3)	58 (45.0)	
Distal	5 (3.4)	0 (0.0)	5 (3.9)	
Branch	1 (0.7)	0 (0.0)	1 (0.8)	
Calcification				0.38
No or mild	2 (1.4)	0 (0.0)	2 (1.6)	
Moderate	22 (14.9)	1 (5.3)	21 (16.3)	
Severe	124 (83.8)	18 (94.7)	106 (82.2)	
Pre-PCI QCA				
Minimum lumen diameter, mm	0.92 (0.69—1.24)	0.90 (0.75—1.09)	0.95 (0.68—1.28)	0.28
Reference vessel diameter, mm	2.50 (2.11—2.92)	2.29 (1.99—2.81)	2.51 (2.12—2.94)	0.60
Diameter stenosis, %	60.4 (51.3—72.4)	61.7 (53.0—74.9)	60.0 (50.2—71.9)	0.39
Lesion length, mm	21.3 (14.9—31.8)	20.9 (17.6—32.4)	21.7 (14.6—31.7)	0.55
Maximum coronary angle at lesion	35 (20—47)	46 (34—65)	33 (19—45)	0.006
PCI procedure				
OA treatment				
Classic crown	128 (86.5)	16 (84.2)	112 (86.8)	0.72
Maximum OA speed				
50,000 rpm	16 (10.8)	3 (15.8)	13 (10.1)	0.44
80,000 rpm	96 (64.9)	15 (78.9)	81 (62.8)	0.21
120,000 rpm	36 (24.3)	1 (5.3)	35 (27.1)	0.04
Frequency of OA ablation, n	6 (5—10)	7 (5—10)	6 (4—9)	0.51
Total OA ablation time, seconds	130 (89—200)	135 (104—190)	130 (87—204)	0.70

LAD indicates left anterior descending artery; LCX, left circumflex artery; RCA, right coronary artery; PCI, percutaneous coronary intervention; QCA; quantitative coronary angiography; OA, orbital atherectomy

### OCT findings

Pre-PCI maximum calcium thickness was thicker in lesions with OA injury than in those without (1120 μm vs. 1000 μm). In addition, more OCT catheter (100% vs. 19%) and GW contact (63% vs. 8%) with normal vessel, larger OCT contact angle (137° vs. 0°), and longer OCT contact length (25 mm vs. 0 mm) and GW contact length (1.8 mm vs. 0 mm) were found in lesions with OA injury than in those without (Table 3). We found no significant differences of other OCT findings, including pre-PCI and post-OA minimum lumen area, reference area, calcium angle and length, and prevalence of calcified nodule between the two groups.

**Table 3:** Optical Coherence Tomography Findings

	Overall (n = 148)	Lesions with OA injury (n = 19)	Lesions without OA injury (n = 129)	*P*	
Pre-PCI MLA, mm2	1.48 (1.09—1.97)	1.48 (0.99—1.86)	1.48 (1.11—2.12)	0.43	
Pre-PCI maximum calcium angle, °	267 (199—360)	234 (195—312)	271 (200—360)	0.53	
Pre-PCI maximum calcium thickness, µm	1030 (890—1140)	1120 (1035—1200)	1000 (890—1113)	0.008	
Pre-PCI calcium length, mm	21 (14—26)	21 (14—27)	16 (9—25)	0.17	
Proximal reference lumen area, mm^2^	6.18 (5.02—7.96)	6.17 (4.10—7.96)	6.20 (5.05—7.96)	0.42	
Distal reference lumen area, mm^2^	4.89 (3.93—6.59)	4.31 (3.96—6.69)	4.97 (3.92—6.58)	0.81	
Mean reference lumen area, mm^2^	5.64 (4.51—7.05)	5.36 (4.17—6.40)	5.66 (4.56—7.07)	0.35	
Calcified nodule, n (%)	32 (21.6)	6 (31.6)	26 (20.2)	0.25	
OCT catheter contact with normal vessel, n (%)	43 (29.1)	19 (100)	24 (18.6)	< 0.001	
GW contact with normal vessel, n (%)	22 (14.9)	12 (63.2)	10 (7.8)	< 0.001	
OCT contact angle, °	0 (0—65)	137 (113—169)	0 (0—0)	< 0.001	
OCT contact length, mm	25.0 (0.0—1.4)	25.0 (3.0—8.0)	0.0 (0.0—0.0)	< 0.001	
GW contact length, mm	0.0 (0.0—0.0)	1.8 (0.0—5.2)	0.0 (0.0—0.0)	< 0.001	
post-OA MLA, mm2	2.06 (1.52—270)	1.65 (1.40—2.31)	2.14 (1.55—2.73)	0.10	

PCI indicates percutaneous coronary intervention; MLA, minimum lumen area; MSA, minimum stent area; OCT, optical coherence tomography; GW, guide wire; OA, orbital atherectomy

### Clinical and OCT characteristics associated to OA injury

**Fig. 2:**
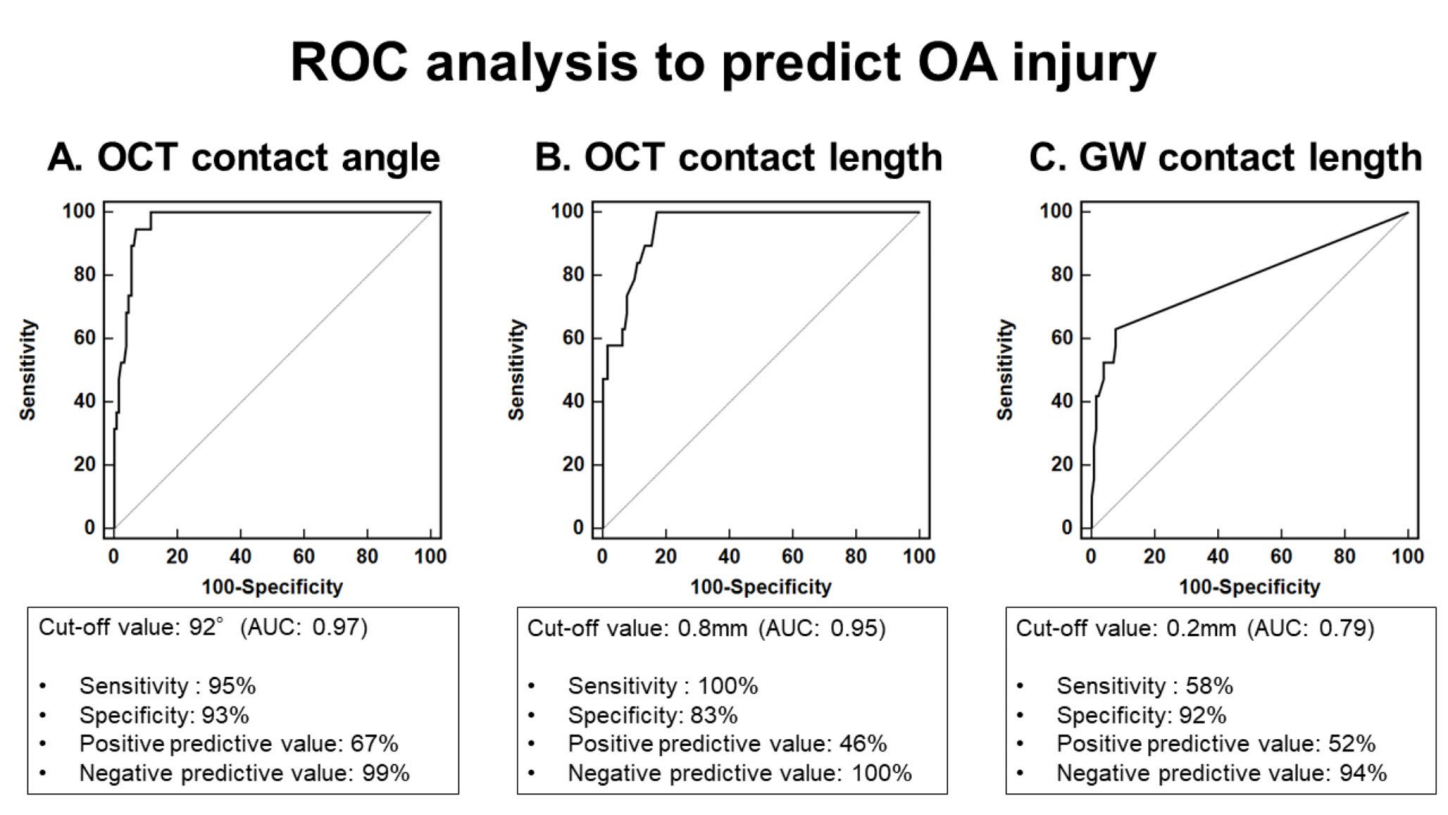
Receiver Operating Characteristic Curves for Measurements of OCT Catheter Contact Angle (A), OCT catheter contact length (B), and guide wire (GW) contact length (C) to Predict Orbital Atherectomy Injury Best cut-off values of OCT catheter contact angle, OCT catheter contact length, and GW contact length for the prediction of OA injury were 92°, 0.8 mm, and 0.2 mm, respectively. AUC, area under the curve

**Fig. 3:**
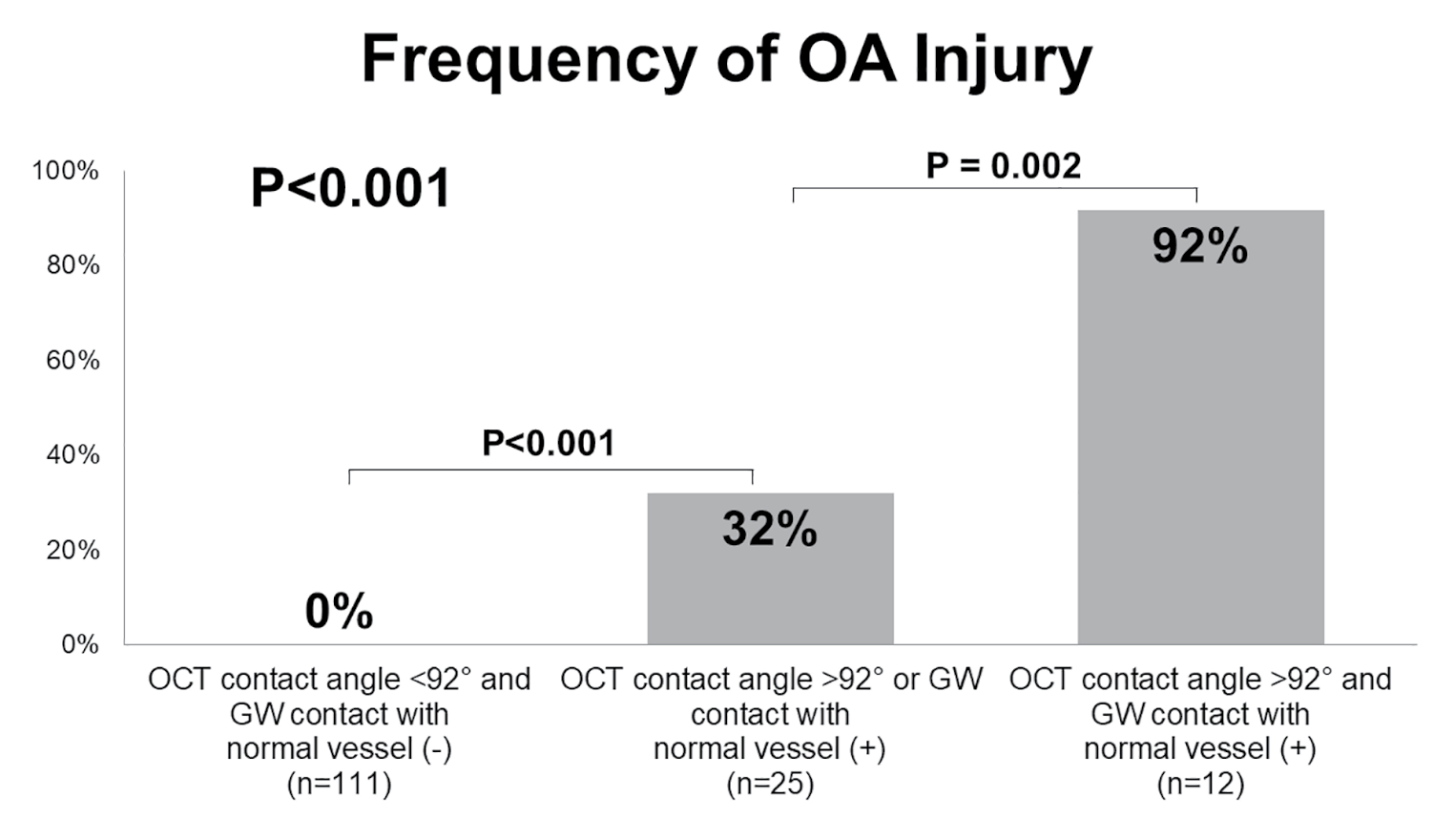
Frequency of OA injury among lesions with or without OCT contact angle > 92° and with or without guide wire (GW) contact with normal coronary artery When considering only OCT contact angle > 92° and the presence of GW contact with normal coronary artery for predicting OA injury, 92% (11/12) of lesions with both findings resulted in OA injury, whereas no OA injury occurred in lesions with neither finding (0/111)

In univariable logistic regression analysis to predict OA injury, OCT contact angle and GW contact with normal vessel were significantly associated with OA injury (Table 4). In ROC analysis for prediction of OA injury at pre-PCI OCT, OCT contact angle of 92 degree was best cut-off value of OA injury with the area under the curve (AUC) as 0.97 (P < 0.001), sensitivity of 0.95, specificity of 0.93, positive predictive value of 0.67, and negative predictive value of 0.99 (Fig. 2A). Furthermore, best cut-off value of OCT contact length and GW contact length for the prediction of OA injury were 0.8 mm and 0.2 mm with the AUC as 0.95 and 0.79, respectively (Fig. 2B C). When considering only OCT contact angle > 92° and the presence of GW contact with normal coronary artery for predicting OA injury, 92% (11/12) of lesions with both findings resulted in OA injury, whereas no OA injury occurred in lesions with neither finding (0/111) (P < 0.001) (Fig. 3).

**Table 4:** Univariable Logistic Regression Analysis of Factors Related to OA Injury

	OR	95% CI	*P*
OCT contact angle, °	1.07	1.03—1.12	0.002
GW contact with normal vessel	11.9	1.07—131.6	0.04
Maximum OA speed less than 120,000 rpm	16.3	0.91—292.1	0.06
Maximum calcium thickness	1.01	0.99—1.01	0.08

OCT indicates optical coherence tomography; GW, guide-wire; OA, orbital atherectomy

## Discussion

In this study, OA injury, defined as the disappearance of both normal vessel intima and media at post-OA due to OA debulking, was found in 19 lesions (13%) and significant relationship was identified between the larger OCT contact angle and the presence of GW contact with normal coronary artery in calcified lesions required OA. Furthermore, both of OCT contact angle > 92° and the presence of GW contact with normal coronary artery was found, more than 90% of lesions resulted in OA injury, whereas no OA injury occurred in lesions with neither. To best our knowledge, this is the first study to assess the association between imaging device or guide-wire bias and coronary artery injury after OA by OCT.

OA system utilizes a 1.25 mm eccentrically mounted diamond-coated crown that rotates in an expanding lateral direction with increasing centrifugal force resulting in a differential sanding of coronary calcification. The OA crown enables both antegrade and retrograde ablation, maintains continuous blood flow while orbiting minimizes the risk of thermal injury to the vessel and produces minuscule particulates with less chance of slow or no-reflow [13]. On the other hand, deeper dissection from OA raises concern for complications, especially in angulated and eccentric lesions. Okamoto N, et al. reported that OA was associated with device-related coronary perforation and dissection compared with RA, whereas device-related complication did not translate to higher rate of post-procedure and 1-year clinical outcome and sub group with OCT imaging showed comparable tissue modification and no statistically significant deep dissection between OA and RA [14].

Past studies reported the relationship between wire or intravascular imaging device bias and atherectomy area, even excessive modification resulted in complication. In IVUS study, Kawaguchi Y, et al. reported that there was no coronary perivascular trauma when the IVUS catheter in contact with the healthy region of the vessel was not found [15]. Additionally, Ye F, et al. reported that touch angle of OCT imaging device to the vessel wall and distance from center of OCT device to media were predictors of RA related intimal dissection by OCT [12]. This is in line with our results. On one hand, IVUS study reported that wire position, not IVUS catheter, was more important for the prediction of optimal RA debulking segments [16]. OA enables “pull-back atherectomy”, which means OA device movement from distal to proximal segment during atherectomy [17], and this movement from distal to proximal segment is similar to OCT or IVUS pull-back imaging compared to RA device movement during atherectomy, which is able to advance only antegradely. Thus, bias of imaging device is potentially more important in OA, especially with pull-back atherectomy than in RA ablation.

In the present study, higher maximum OA speed was inversely associated with OA injury though theoretically higher speed OA is associated with vessel injury. In this study, we performed OCT imaging and angiogram after OA and assessed whether there was complication (vessel injury, severe dissection, slow/no-reflow, etc.) or not. When severe complication aforementioned were not observed, then we proceed to higher speed OA. In other word, when we found vessel injury or other complication after initial lower speed OA, we did not go to the next step of higher speed OA. In this “step-by-step strategy”, therefore, OA injury in higher speed OA were less frequent than in lower speed OA.

To predict severe complications, including coronary perforation is challenging. Device induced coronary perforation was 0.3–2.1% after RA and 0.9–1.6% after OA, respectively as previously reported [2, 14, 18–20]. Past studies have reported factors associated with complication with RA [19, 20]. Wang YH, et al. reported that unintended and unnoticed bias cutting into noncalcified plaque or through calcified vessel wall was an important cause of coronary perforation after RA [20]. Similarly, in case report of PCI with OA, wire bias was reported as an important predictive factor related to the coronary artery injury [6]. Although treatment options when we found imaging or wire bias is still unclear, ablation with pull-back atherectomy only in OA might be safer than both antegrade and pull-back atherectomy [17]. If possible, to cross the wire to other side branch located at distal from target lesion may make the wire bias safer or more effective to ablate calcified plaque. In the present study, we found 7.4% of vessel injury at plaque side (not normal segment) and most of them were at lipid plaque adjacent to calcification. To avoid excessive ablation, in not only normal vessel segment but also plaque side, would be also important in terms of prevention of severe complication, including coronary perforation. Although there were no lesions with coronary perforation and no patients underwent covered stent implantation in this study population, excessive ablation of the normal segment has a potential risk of coronary perforation [21]. The OA is able to treat severely calcified lesions effectively. However, operators need to pay more attention about the wire or device position to avoid critical complications by using intravascular imaging, including OCT, which can provide not only calcified plaque information but also device and wire bias.

### Limitations

There were several limitations in the present study. First, in this retrospective observational study, PCI procedure, including OA treatment (i.e. maximum OA speed), was left to the operator’s discretion. Therefore, selection bias was inevitable. Second, there were small number of population and OA injury. Thus, we did not have enough statistical power for multivariable logistic regression analysis to assess predictors of OA injury, whereas univariable assessment showed only OCT contact angle and the presence of GW contact with normal coronary artery were statistically significant. Third, we excluded lesions with anticipated difficulty in advancing the OCT catheter, such as lesions with severe narrowing, tortuosity or severe calcification. Fourth, it is unclear whether OA injury is associated with severe complication, including coronary perforation. However, composition of coronary artery without intima and media, even without adventitia, is nearly equivalent to pseudoaneurysm and was thought to be potentially associated with coronary perforation when inadequate treatment was performed. Furthermore, past case study reported that debulking of normal vessel resulted in aneurysmal formation of coronary artery [22]. Therefore, we need to pay more attention these potentially high-risk imaging findings.

## Conclusion

Pre-PCI OCT findings, such as catheter contact angle > 92° and guide-wire contact to the normal coronary artery, were associated with post-OA coronary artery injury.
